# 2-(2-Hydroxy­ethyl)-3-[(2-hydroxy­ethyl)imino]isoindolin-1-one

**DOI:** 10.1107/S1600536809030487

**Published:** 2009-08-08

**Authors:** Jiří Urban, Jiří Ludvík, Jan Fábry, Michal Dušek, Karla Fejfarová

**Affiliations:** a J. Heyrovsky Institute of Physical Chemistry, Academy of Sciences of the Czech Republic, Dolejškova 3, 182 23 Praha 8, Czech Republic; b Institute of Physics of the Czech Academy of Sciences, Na Slovance 2, 182 21 Praha 8, Czech Republic

## Abstract

In the crystal structure of the title compound, C_12_H_14_N_2_O_3_, mol­ecules are packed into layers parallel to (100). Each layer contains centrosymmetric dimers formed by a pair of strong O—H⋯N hydrogen bonds with an *R*_2_^2^(10) motif, while strong O—H⋯O hydrogen bonds forming *C*(10) chains connect mol­ecules into a two-dimensional network. Additional stabilization is supplied by weak C—H⋯O hydrogen bonds and weak π–π stacking inter­actions with centroid–centroid distances in the range 3.4220 (7)–3.9616 (7) Å.

## Related literature

For background to the chemical and electrochemical properties of aromatic dicarbonyl compounds, see: Zuman (2004 ▶). For the use of reactions between phthalaldehyde and nucleophiles for the fluorimetric determination of amino acids, see: Roth (1971 ▶); For other structures isolated from systems in which kolamine was reacted with phthalaldehyde, see: Urban (2007*a* ▶, 2007*b* ▶). For hydrogen bonding and graph-set motifs, see: Desiraju & Steiner (1999 ▶); Etter *et al.* (1990 ▶). For the extinction correction, see: Becker & Coppens (1974 ▶).
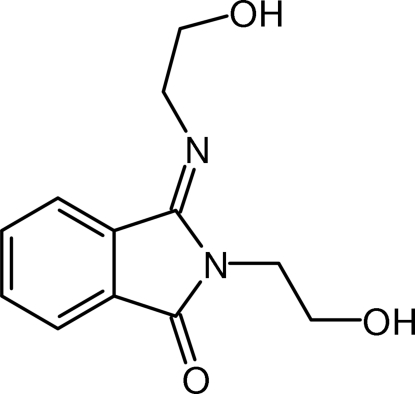


## Experimental

### 

#### Crystal data

C_12_H_14_N_2_O_3_
*M* *_r_* = 234.3
Orthorhombic, 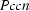

*a* = 19.04539 (11) Å
*b* = 7.14668 (5) Å
*c* = 15.71068 (9) Å
*V* = 2138.40 (5) Å^3^
*Z* = 8
Cu *K*α radiation
μ = 0.88 mm^−1^
*T* = 120 K
0.64 × 0.11 × 0.05 mm

#### Data collection

Oxford Diffraction Gemini diffractometer
Absorption correction: multi-scan (*CrysAlis RED*; Oxford Diffraction, 2005 ▶) *T*_min_ = 0.547, *T*_max_ = 0.916
40508 measured reflections
1829 independent reflections
1676 reflections with *I* > 3σ(*I*)
*R*_int_ = 0.032

#### Refinement

*R*[*F* ^2^ > 2σ(*F* ^2^)] = 0.029
*wR*(*F* ^2^) = 0.088
*S* = 3.44
1829 reflections
161 parameters
H atoms treated by a mixture of independent and constrained refinement
Δρ_max_ = 0.21 e Å^−3^
Δρ_min_ = −0.19 e Å^−3^

### 

Data collection: *CrysAlis CCD* (Oxford Diffraction, 2005 ▶); cell refinement: *CrysAlis RED* (Oxford Diffraction, 2005 ▶); data reduction: *CrysAlis RED*; program(s) used to solve structure: *SIR97* (Altomare *et al.*, 1997 ▶); program(s) used to refine structure: *JANA2006* (Petříček *et al.*, 2006 ▶); molecular graphics: *PLATON* (Spek, 2009 ▶); software used to prepare material for publication: *JANA2006*.

## Supplementary Material

Crystal structure: contains datablocks global, I. DOI: 10.1107/S1600536809030487/lh2865sup1.cif

Structure factors: contains datablocks I. DOI: 10.1107/S1600536809030487/lh2865Isup2.hkl

Additional supplementary materials:  crystallographic information; 3D view; checkCIF report

## Figures and Tables

**Table 1:** Hydrogen-bond geometry (Å, °)

*D*—H⋯*A*	*D*—H	H⋯*A*	*D*⋯*A*	*D*—H⋯*A*
O3—H1O3⋯N2^i^	0.85 (2)	1.97 (2)	2.8145 (12)	176 (2)
O2—H1O2⋯O3^ii^	0.86 (2)	1.94 (2)	2.7903 (12)	173 (2)
C9—H2C9⋯O3^i^	0.97	2.60	3.4074 (14)	141
C4—H1C4⋯O1^iii^	0.93	2.37	3.2553 (15)	159
C5—H1C5⋯O2^iii^	0.93	2.51	3.4239 (15)	167

Symmetry codes: (i) 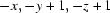; (ii) 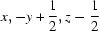; (iii) 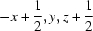.

**Fig. 1.**
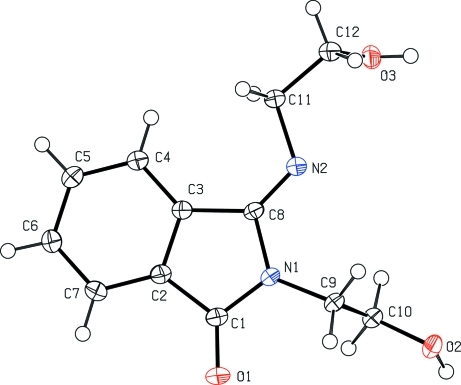
The title molecule with the atomic numbering scheme. The displacement ellipsoids are shown at the 50% probability level.

**Fig. 2.**
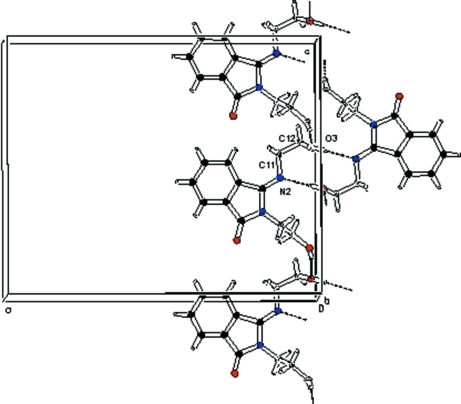
Part of the crystal structure showing a R22(10) motif (Etter et al., 1990) involving the O—H···N hydrogen bonds (dashed lines) forming centrosymmetric dimers.

**Fig. 3.**
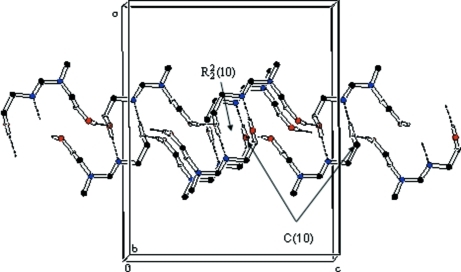
Part of the crystal structure viewed along the axis b showing the R22(10) and C(10) hydrogen-bond motifs formed by O—H···N and O—H···O hydrogen bonds (dashed lines), respectively. The arrows point to the centre of the ring R22(10) and to the extremal atoms of the chain C(10). Only those atoms that are relevant to the hydrogen bonded graph sets are shown.

**Fig. 4.**
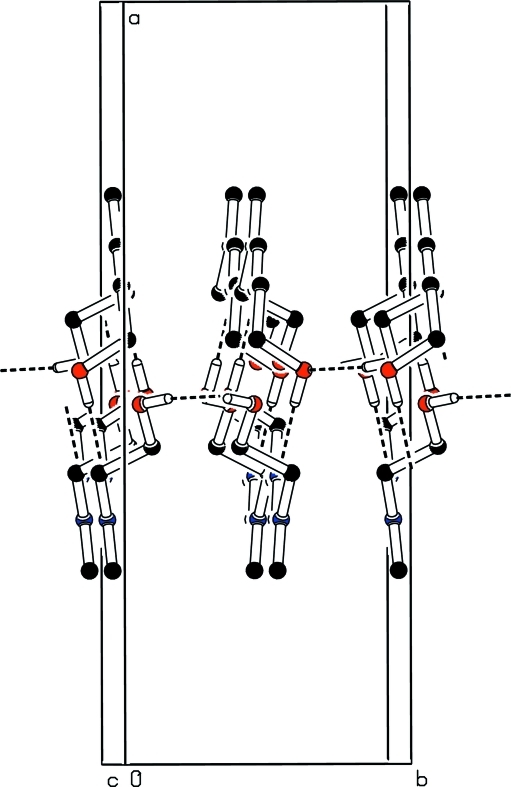
Part of the crystal structure showing the O—H···O hydrogen-bonds which interconnect the structure in the b axis direction. Only those atoms that are relevant to the graph sets formation are showm.

**Fig. 5.**
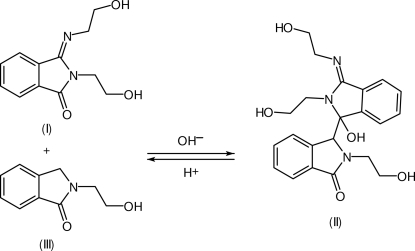
The suggested reaction scheme of the title compound. (II): 1-(2-Hydroxyethyl)imino-2-(2-hydroxyethyl)-3-hydroxy -3-(2'-(2-hydroxyethyl)-2',3'-dihydro-1'H-benzo[c]pyrrol- 1'-one-3-yl)-2,3-dihydro-1H-benzo[c]pyrrole (III): 2-(2-hydroxyethyl)-2,3-dihydro-1H-benzo[c] pyrrol-1-one (Urban et al., 1997a)

## supplementary crystallographic information

### Comment

In the course of the authors' investigations on the chemical and electrochemical properties of aromatic dicarbonyl compounds (Zuman, 2004) the reactivity of phthalaldehyde with nucleophiles has been studied. The reaction between phthalaldehyde and nucleophiles is important because namely the reaction of phthalaldehyde with amino acids is used for the fluorimetric determination of the latter substances. The fluorescence is substantially enhanced if another, stronger nucleophile is added in excess to phthalaldehyde prior to its reaction with an amino acid (Roth, 1971). Despite of the practical application of this reaction the chemical mechanism of this a rather complex process is still not fully understood. In order to elucidate the reaction steps we started to study the reactions between phthalaldehyde and a stronger nucleophile [2-aminoethanol (kolamine)] before adding an amino acid.

The interaction between phthalaldehyde and a nucleophile seems to be crucial because it is strongly dependent on a medium, on the sequence of the added chemicals (kolamine prior to phthalaldehyde or *vice versa*) as well as on the way, *e.g.* its speed, of their mixing as follows from our preliminary studies (Urban *et al.*, 2007*a*,b). The aim of this study is to investigate the reaction pathway by isolation of some intermediate products before adding an amino acid.

As the first studied nucleophile we have used 2-aminoethanol (kolamine). A previously applied procedure (Urban *et al.* 2007*a*,b) involving the addition reaction in non-aqueous acetonitrile when kolamine was added dropwise led to the isolation of two products. In contrast to these previous studies the reaction presented here proceeded in 0.1 *M* HCl and yielded the title compound that is different from those described by Urban *et al.* (2007*a*,b; see Fig. 5). The study that aims to explain this difference in the reactions paths in various evironments is under progress as well as the study of the detailed reaction pathway of the subsequent reaction with an amino acid.

The molecular structure of the title compound is shown in Fig. 1. In the crystal structure, the most important intermolecular interactions between the molecules are strong (Desiraju & Steiner, 1999) O—H···N and O—H···O hydrogen bonds (Table 1). The O3—H3···N2^i^ [symmetry codes as in Table 1] hydrogen bonds are involved in the formation of dimers with the graph set motif *R*_2_^2^(10) (Etter *et al.*, 1990; Fig. 2, Fig. 3). These dimers are located on crystallographic inversion centers. Each such a dimer is interconnected to another one displaced by 0, 1/2, 1/2 *via* O—H···O hydrogen bond with the motif C(10) (Fig. 3). These chains together with the above mentioned dimers are interconnected by other symmetry-equivalent O—H···O hydrogen bonds and form a two- dimensional pattern (Fig. 4) that is parallel to (100). There are also week C—H···O hydrogen bonds and it is interesting that the carbonyl oxygen O1 is involved in a weak C—H···O hydrogen bonding.

There are also π–π stacking interactions between the aromatic rings of the title molecule as indicates their stacking along the *b* axes in the approximate distance *b*/2=3.57334 (3) Å. Specifically, the distances between the centroids of the pyrrole rings equal to 3.4220 (7) and 3.8779 (7) Å for the rings displaced by 1/2 - *x*, 3/2 - *y*, *z* and 1/2 - *x*,1/2 - *y*, *z*, respectively. The distances between the centroids of the pyrrole and benzene rings equal to 3.8705 (7) and 3.9616 (7) Å for the benzene rings displaced by 1/2 - *x*, 3/2 - *y*, *z* and 1/2 - *x*, 1/2 - *y*, *z*, respectively.

The attempts at crystallizations of other isolated compounds failed and therefore their molecular structures were assigned by ^1^H and ^13^C NMR spectrometry (Fig. 5). The spectra were taken on the NMR spectrometer Varian 300 MHz at frequencies 229.970 and 75.434 MHz for ^1^H and ^13^C, respectively.

### Experimental

3 ml (50 mmol) of 2-aminoethanol was dissolved in 247.5 ml of 0.1 *M* HCl. This solution was poured to other solution of of phthalaldehyde (660 mg, 4.9 mmol) in 15 ml of ethanol. The mixture was stirred for 4 h. Then it was filtered and evaporated to dryness under reduced pressure. The residue was dissolved in CHCl_3_, the solution was washed with water, dried and evaporated to dryness. The residue (870 mg) was chromatographed on a column of 110 g of silica gel in CHCl_3_-ethanol (9:1 *v*/*v*). The column chromatography afforded 123 mg of the title compound (I) (see Fig. 5) and 192 mg of the compound (II) among other compounds in undefinable mixtures. The title compound was dissolved in about 4 ml of warm CHCl_3_ and the same volume of toluene was added. After one week light-brown, 2–3 mm long needle crystals were separated. The attempts to obtain single crystals of (II) was not successful despite of all efforts. (From the mixtures of chloroform - ethanol (1:1 *v*/*v*) as well as chloroform - toluene- n-hexane (2:1:1 *v*/*v*) microcrystallne form has been obtained at best. The structure of (II) was inferred from the NMR spectra ^1^H and ^13^C. The isolated compound (II) is not stable, it decomposes, especially in acid medium (pH~4), into the compound (I) and 2-(2-hydroxyethyl)-2,3-dihydro-1*H*-benzo[*c*]pyrrol-1-one (III) - see Scheme 2. The NMR spectrum of (III) is the same as that of 2-(2-hydroxyethyl)-2,3-dihydro-1*H*-benzo[*c*]pyrrol-1-one the crystal structure of which has already been determined (Urban, 1997*a*).

### Refinement

All the hydrogen atoms could be distinguished in the electron-density difference maps. Furthermore, all the hydrogen atoms could have been refined yielding good geometry. Nevertheless, all the H atoms attached to the carbon atoms have been constrained in the riding motion approximation while the coordination parameters of the hydroxyl H atoms that are involved in the strong hydrogen bonds have been freely refined. The values of the constraints are: C_aryl_—H=0.93, C_methylene_—H=0.97 Å. *U*_iso_H=1.2*U*_eq_C_aryl_/C_methylene_/O_hydroxyl_.

### Crystal data

**Table:**

C_12_H_14_N_2_O_3_	*D*_x_ = 1.455 Mg m^−^^3^
*M**_r_* = 234.3	Melting point = 373–375 K
Orthorhombic, *P**c**c**n*	Cu *K*α radiation, λ = 1.54184 Å
Hall symbol: -P 2ab 2ac	Cell parameters from 26197 reflections
*a* = 19.04539 (11) Å	θ = 2.8–65.3°
*b* = 7.14668 (5) Å	µ = 0.88 mm^−^^1^
*c* = 15.71068 (9) Å	*T* = 120 K
*V* = 2138.40 (5) Å^3^	Needle, brown
*Z* = 8	0.64 × 0.11 × 0.05 mm
*F*(000) = 992	

### Data collection

**Table:**

Oxford Diffraction Gemini diffractometer	1829 independent reflections
Radiation source: Ultra (Cu) X-ray Source	1676 reflections with *I* > 3σ(*I*)
mirror	*R*_int_ = 0.032
Detector resolution: 10.3784 pixels mm^-1^	θ_max_ = 65.4°, θ_min_ = 2.8°
ω scans	*h* = −21→22
Absorption correction: multi-scan (*CrysAlis RED*; Oxford Diffraction, 2005)	*k* = −7→8
*T*_min_ = 0.547, *T*_max_ = 0.916	*l* = −18→18
40508 measured reflections	

### Refinement

**Table:**

Refinement on *F*^2^	Secondary atom site location: difference Fourier map
*R*[*F*^2^ > 2σ(*F*^2^)] = 0.029	Hydrogen site location: difference Fourier map
*wR*(*F*^2^) = 0.088	H atoms treated by a mixture of independent and constrained refinement
*S* = 3.44	Weighting scheme based on measured s.u.'s *w* = 1/[σ^2^(*I*) + 0.0004*I*^2^]
1829 reflections	(Δ/σ)_max_ = 0.001
161 parameters	Δρ_max_ = 0.21 e Å^−^^3^
0 restraints	Δρ_min_ = −0.19 e Å^−^^3^
50 constraints	Extinction correction: B–C type 1 Lorentzian isotropic (Becker & Coppens, 1974)
Primary atom site location: structure-invariant direct methods	Extinction coefficient: 3600 (400)

### Fractional atomic coordinates and isotropic or equivalent isotropic displacement parameters (Å^2^)

**Table:**

	*x*	*y*	*z*	*U*_iso_*/*U*_eq_	
O3	0.01643 (4)	0.37865 (12)	0.58348 (5)	0.0249 (3)	
O2	0.02777 (4)	0.42770 (13)	0.19272 (5)	0.0254 (3)	
N2	0.12251 (5)	0.53455 (13)	0.46306 (6)	0.0189 (3)	
N1	0.17906 (5)	0.53362 (14)	0.33037 (6)	0.0195 (3)	
O1	0.26033 (5)	0.52000 (13)	0.22084 (5)	0.0295 (3)	
C8	0.17913 (6)	0.52397 (15)	0.41996 (7)	0.0167 (4)	
C10	0.08334 (6)	0.38375 (17)	0.24969 (7)	0.0217 (3)	
C3	0.25448 (7)	0.50383 (14)	0.44476 (7)	0.0168 (3)	
C2	0.29391 (7)	0.50185 (15)	0.36982 (7)	0.0182 (4)	
C6	0.40008 (7)	0.47776 (16)	0.44756 (8)	0.0228 (4)	
C9	0.11818 (6)	0.56558 (17)	0.27593 (7)	0.0209 (4)	
C4	0.28841 (6)	0.49167 (15)	0.52267 (8)	0.0202 (4)	
C12	0.05695 (6)	0.54373 (18)	0.59873 (7)	0.0229 (4)	
C11	0.12770 (6)	0.52705 (16)	0.55650 (7)	0.0198 (4)	
C7	0.36643 (6)	0.49080 (15)	0.36957 (8)	0.0208 (4)	
C5	0.36134 (7)	0.47820 (16)	0.52273 (8)	0.0228 (4)	
C1	0.24599 (6)	0.51820 (16)	0.29654 (7)	0.0202 (4)	
H1c10	0.117323	0.30469	0.22104	0.026*	
H2c10	0.064404	0.321905	0.299633	0.026*	
H1c9	0.132549	0.633958	0.225521	0.0251*	
H2c9	0.084468	0.643488	0.305694	0.0251*	
H1c12	0.063213	0.560506	0.659524	0.0275*	
H2c12	0.032334	0.651588	0.576051	0.0275*	
H1c11	0.158048	0.626955	0.576323	0.0238*	
H2c11	0.149675	0.410391	0.573408	0.0238*	
H1c6	0.448764	0.468686	0.44947	0.0273*	
H1c4	0.263062	0.492517	0.573256	0.0243*	
H1c7	0.391612	0.492081	0.318854	0.025*	
H1c5	0.384849	0.469256	0.57446	0.0273*	
H1o3	−0.0255 (9)	0.409 (2)	0.5715 (10)	0.0373*	
H1o2	0.0205 (8)	0.335 (2)	0.1590 (10)	0.0381*	

### Atomic displacement parameters (Å^2^)

**Table:**

	*U*^11^	*U*^22^	*U*^33^	*U*^12^	*U*^13^	*U*^23^
O3	0.0162 (5)	0.0353 (5)	0.0231 (4)	0.0000 (4)	−0.0010 (3)	0.0066 (3)
O2	0.0200 (4)	0.0346 (5)	0.0217 (4)	0.0008 (4)	−0.0064 (3)	−0.0041 (4)
N2	0.0179 (5)	0.0237 (5)	0.0150 (5)	0.0000 (4)	0.0003 (4)	0.0005 (4)
N1	0.0172 (5)	0.0284 (6)	0.0128 (5)	0.0002 (4)	−0.0020 (4)	0.0000 (4)
O1	0.0266 (5)	0.0491 (6)	0.0130 (4)	0.0007 (4)	0.0017 (3)	−0.0006 (3)
C8	0.0195 (6)	0.0183 (6)	0.0123 (6)	−0.0009 (4)	−0.0015 (5)	0.0000 (4)
C10	0.0191 (6)	0.0295 (7)	0.0163 (6)	0.0012 (5)	−0.0023 (4)	0.0000 (4)
C3	0.0184 (6)	0.0168 (6)	0.0152 (6)	−0.0005 (4)	0.0009 (5)	−0.0003 (4)
C2	0.0201 (7)	0.0186 (6)	0.0159 (6)	0.0005 (4)	0.0000 (4)	−0.0010 (4)
C6	0.0179 (7)	0.0268 (7)	0.0235 (7)	0.0024 (5)	−0.0006 (5)	−0.0006 (5)
C9	0.0195 (6)	0.0293 (7)	0.0140 (6)	0.0014 (5)	−0.0030 (4)	0.0022 (5)
C4	0.0205 (7)	0.0242 (6)	0.0161 (6)	0.0005 (4)	0.0001 (5)	0.0005 (4)
C12	0.0221 (6)	0.0302 (7)	0.0163 (6)	0.0018 (5)	0.0012 (5)	0.0003 (4)
C11	0.0194 (6)	0.0264 (7)	0.0137 (6)	−0.0005 (4)	0.0001 (4)	0.0016 (4)
C7	0.0202 (7)	0.0228 (6)	0.0194 (7)	0.0013 (4)	0.0034 (5)	−0.0015 (4)
C5	0.0227 (7)	0.0271 (7)	0.0185 (6)	0.0009 (5)	−0.0036 (5)	0.0011 (4)
C1	0.0220 (6)	0.0243 (6)	0.0143 (6)	−0.0007 (5)	0.0003 (5)	−0.0010 (4)

### Geometric parameters (Å, °)

**Table:**

O3—C12	1.4300 (15)	C4—C5	1.3923 (18)
O2—C10	1.4213 (13)	C12—C11	1.5067 (16)
N2—C8	1.2756 (15)	O3—H1o3	0.848 (17)
N2—C11	1.4724 (15)	O2—H1o2	0.859 (16)
N1—C8	1.4092 (15)	C10—H1c10	0.9700
N1—C9	1.4587 (14)	C10—H2c10	0.9700
N1—C1	1.3856 (15)	C6—H1c6	0.9300
O1—C1	1.2204 (14)	C9—H1c9	0.9700
C8—C3	1.4940 (17)	C9—H2c9	0.9700
C10—C9	1.5163 (17)	C4—H1c4	0.9300
C3—C2	1.3966 (16)	C12—H1c12	0.9700
C3—C4	1.3867 (17)	C12—H2c12	0.9700
C2—C7	1.3833 (17)	C11—H1c11	0.9700
C2—C1	1.4737 (16)	C11—H2c11	0.9700
C6—C7	1.3860 (17)	C7—H1c7	0.9300
C6—C5	1.3925 (17)	C5—H1c5	0.9300
			
C8—N2—C11	118.06 (10)	O2—C10—H2c10	109.47
C8—N1—C9	126.45 (9)	C9—C10—H1c10	109.47
C8—N1—C1	112.22 (9)	C9—C10—H2c10	109.47
C9—N1—C1	121.26 (9)	H1c10—C10—H2c10	110.98
N2—C8—N1	121.76 (10)	N1—C9—H1c9	109.47
N2—C8—C3	132.79 (10)	N1—C9—H2c9	109.47
N1—C8—C3	105.44 (9)	C10—C9—H1c9	109.47
O2—C10—C9	107.92 (9)	C10—C9—H2c9	109.47
C8—C3—C2	107.31 (10)	H1c9—C9—H2c9	106.93
C8—C3—C4	133.15 (11)	O3—C12—H1c12	109.47
C2—C3—C4	119.53 (11)	O3—C12—H2c12	109.47
C3—C2—C7	122.67 (11)	C11—C12—H1c12	109.47
C3—C2—C1	108.95 (10)	C11—C12—H2c12	109.47
C7—C2—C1	128.36 (11)	H1c12—C12—H2c12	108.84
C7—C6—C5	120.31 (12)	N2—C11—H1c11	109.47
N1—C9—C10	111.90 (10)	N2—C11—H2c11	109.47
C3—C4—C5	118.03 (11)	C12—C11—H1c11	109.47
O3—C12—C11	110.10 (9)	C12—C11—H2c11	109.47
N2—C11—C12	112.09 (9)	H1c11—C11—H2c11	106.72
C2—C7—C6	117.58 (11)	C3—C4—H1c4	120.79
C6—C5—C4	121.87 (11)	C5—C4—H1c4	121.18
N1—C1—O1	125.37 (11)	C6—C5—H1c5	119.07
N1—C1—C2	106.05 (9)	C4—C5—H1c5	119.06
O1—C1—C2	128.58 (11)	C7—C6—H1c6	119.61
C12—O3—H1o3	109.5 (10)	C5—C6—H1c6	120.07
C10—O2—H1o2	109.8 (10)	C2—C7—H1c7	121.12
O2—C10—H1c10	109.47	C6—C7—H1c7	121.30

### Hydrogen-bond geometry (Å, °)

**Table:**

*D*—H···*A*	*D*—H	H···*A*	*D*···*A*	*D*—H···*A*
O3—H1O3···N2^i^	0.849 (17)	1.968 (17)	2.8145 (12)	175.8 (16)
O2—H1O2···O3^ii^	0.860 (15)	1.935 (15)	2.7903 (12)	173.0 (15)
C9—H2C9···O3^i^	0.97	2.60	3.4074 (14)	141
C4—H1C4···O1^iii^	0.93	2.37	3.2553 (15)	159
C5—H1C5···O2^iii^	0.93	2.51	3.4239 (15)	167

Symmetry codes: (i) −*x*, −*y*+1, −*z*+1; (ii) *x*, −*y*+1/2, *z*−1/2; (iii) −*x*+1/2, *y*, *z*+1/2.
